# Real-World Data Presenting the Descriptive Analysis of the Use of Tyrosine Kinase Inhibitors (TKIs) Among Metastatic Non–Small-Cell Lung Cancer (mNSCLC) Patients in Qatar: A Nationwide Retrospective Cohort Study

**DOI:** 10.1177/11795549241272490

**Published:** 2024-10-15

**Authors:** Rawan Dawoud, Harman Saman, Kakil Rasul, Farah Jibril, Arwa Sahal, Randa Al-Okka, Yaser Mahfouz, Nabil E. Omar, Anas Hamad, Reyad Mohsen, Aladdin Kanbour, Naim Battikh, Prem Chandra, Shereen Elazzazy

**Affiliations:** 1 Department of Pharmacy, T﻿he National Center of Cancer Care and Research, Hamad Medical Corporation, Doha, Qatar; 2 Department of Pulmonary Medicine, Hazm Mebaireek General Hospital, Hamad Medical Corporation, Doha, Qatar; 3 Department of Medical Oncology, T﻿he National Center of Cancer Care and Research, Hamad Medical Corporation, Doha, Qatar; 4 College of Pharmacy, QU Health, Qatar University, Qatar; 5 Department of Medicine, Hamad General Hospital, Hamad Medical Corporation, Doha, Qatar; 6 Department of Medical Research, Academic Health System, Hamad Medical Corporation, Doha, Qatar

**Keywords:** Lung cancer, tyrosine kinase inhibitors, non–small-cell lung cancer, epidermal growth factor, anaplastic lymphoma kinase

## Abstract

**Background:**

There has been significant improvement in treating metastatic non–small-cell lung cancer (mNSCLC) over the past 2 decades. The aim of this study is to describe the use of tyrosine kinase inhibitors (TKIs) in Qatar. This study focuses on the objective response rate (ORR) and reported adverse drug events (ADEs) of TKIs used for the management of patients with mNSCLC.

**Methods:**

This is a descriptive retrospective cohort study. All non–small-cell lung cancers (NSCLCs) with epidermal growth factor receptor (EGFR) or anaplastic lymphoma kinase (ALK) mutations who received TKIs between 2015 and 2019 in Qatar were included. The TKIs used during this period include EGFR inhibitors such as afatinib, erlotinib, gefitinib, and osimertinib and ALK inhibitors such as alectinib and crizotinib. The response on each TKI was identified by reporting the ORR (as the sum of the complete response [CR] and the partial response [PR]), in addition stable disease (SD) and disease progression (DP) were reported. While ADEs were reported using the National Cancer Institute’s Common Terminology Criteria for Adverse Events (NCI-CTCAE).

**Results:**

A total of 63 patients were included, of which 36 cases (57.1%) expressed EGFR mutation, and 27 patients (42.9%) expressed ALK rearrangement. The ORR in EGFR inhibitors was as follows: osimertinib 40%, gefitinib 33%, afatinib 22%, and erlotinib 18%. However, the response to the ALK-targeted therapy was 43% with alectinib and 40% with crizotinib. A total of 112 ADEs were reported. They were distributed as 63.4% (71 of 112) with the anti-EGFR and 36.6% (41 of 112) ADEs with the ALK inhibitors. In the anti-EGFR group, the most common types of ADEs were dermatological toxicity 30%, whereas, in the anti-ALK group, gastrointestinal toxicity was the most common (29%).

**Conclusions:**

The EGFR-targeted and ALK-targeted therapies appear to have acceptable clinical response rate and safety profile in our population. Close and frequent monitoring of adverse events is advised to ensure a good quality of life and prevent serious complications.

## Introduction

As Qatar considerably depends on migrant labors, so the number of populations varies from one season to other, according to the planning and statistic authority in Qatar till end of February 2024, the total population is 3 119 589. The major ethnicity is presented with the South Asians (Indians, Nepalese, Bangladeshis, Sri Lanka, and Pakistanis); they represent 1.1 million in 2024.^1^ Health services and treatment are available for all residents in Qatar, where cancer therapy is offered as free of charges for all citizens and residents. In addition, in-house next-generation sequencing (NGS) testing is provided for all citizens and residents in Qatar who are diagnosed with cancer against no fees.

The STEPWise WHO tobacco survey 2013 focused mainly on male smoking (all tobacco) rates in Qatar, which showed high incidence of 31.9%. This urged the need for extended efforts to reduce smoking figures.^2^

Lung cancer (LC) remains one of the most common types of cancer and the leading causes of death worldwide.^3,4^ It accounted for 1.8 million deaths, 18.4% of total mortality, which is the largest mortality proportion among cancer patients in 2018 according to the International Agency for Research on Cancer (IARC).^5,6^ As per Qatar National Cancer Registry 2013 to 2015, LC comes in the sixth place among the most common cancer types in Qatar. Histologically, LC is broadly classified into non–small-cell lung cancer (NSCLC; 85%) of patients and small-cell lung cancer (SCLC; 15%).^7^ The NSCLC is commonly not diagnosed until advanced stages of the disease.^6^ Before the era of precision medicine and targeted therapy, NSCLC management was mainly by intravenous conventional platinum-based chemotherapy as the mainstay therapy. At that time, the 5-year overall survival rate for patients with mNSCLC was less than 5%. However, in the past 2 decades, significant developments have been introduced to the science of NSCLC. The improved understanding of NSCLC’s biology and the targeting of somatic mutations has shifted the paradigm of the treatment.^7^ These molecular alterations that predict response to treatment are present in approximately 20% to 60% of patients with NSCLC depending on the geographical area.^8^ The most common 2 targetable driver mutations in mNSCLC with adenocarcinoma histology are the epidermal growth factor receptor (EGFR) and the anaplastic lymphoma kinase (ALK) with incidence rate of 10% to 15% and 3% to 7%, respectively.^9^ In Qatar, clinical practice and cancer care guidelines of NSCLC are adopted from the international guidelines such as [the American Society of Clinical Oncology (ASCO), the Europian Society of Medical Oncology (ESMO), and the National Comprehensive Cancer Network (NCCN)]. Following the international guidelines, the standard of care in Qatar is to manage metastatic adenocarcinoma NSCLC patients with platinum-based regimens plus pemetrexed as the first line, followed by taxanes-based regimen on progression and then to target the EGFR or ALK mutations with the appropriate available formulary tyrosine kinase inhibitor (TKI).

Nowadays, targeted therapy have replaced chemotherapy as the first-line management, such as EGFR inhibitors (eg, erlotinib, gefitinib), and NTRK/ROS1 inhibitors (eg, entrectinib).^10^ In addition, a meta-analysis published in 2013, in which an EGFR TKI was compared with platinum-based chemotherapy; PFS was significantly prolonged in favor to the TKIs group (hazard ratio [HR] = 0.43; 95% confidence interval [CI] = 0.38-0.49). Lower rates of adverse effects and better symptom control were also reported in favor of the EGFR inhibitors.^11^

With more focus on TKIs, we need to discuss receptor tyrosine kinases (RTKs). They are a class of cell surface receptors that play a critical role in cellular signaling pathways. The TKIs are drugs developed to block RTKs acting on the cellular signaling pathway. Currently, only a few of numerous identified molecular targets are relevant in clinical practice, although the list of targetable mutations is growing, as several potent TKIs have moved from phase II to phase III clinical trials.^7^ Even more excitingly, new TKIs continue to graduate from clinical trial to clinical practice and become part of national and international guidelines. The use of TKIs in stage 4 NSCLC has become well established in the past decade, both as upfront strategy and as second-line strategy after progression from platinum-based chemotherapy.^12^

The most important targeted treatment options in NSCLC include the epidermal growth factor receptor inhibitors (EGFRis), the vascular endothelial growth factor receptor inhibitors (VEGFRis), and the echinoderm microtubule–associated protein-like 4–anaplastic lymphoma kinase (EML4-ALK) fusion gene inhibitors.^8^ Other rare mutations are KRAS, ROS1, RET, and HER2. Many drugs targeting these pathways have been developed and shown clinical benefits with improvements in both overall and progression-free survivals for patients with advanced or metastatic disease compared with cytotoxic chemotherapy.^13^ Studying the clinical effects of TKIs is critical to improving our understanding of these therapies and optimizing their use in cancer treatment in our population and optimizing health outcomes.

Researchers and clinicians at the National Center for Cancer Care and Research (NCCCR) in Qatar are working collaboratively to fill in the knowledge gap caused by scarcity of data in relation to the efficacy and safety of TKIs use among our patients with advanced NSCLC.

The objectives of this descriptive retrospective study are to overview the therapeutic clinical outcomes and adverse effects of TKIs used to manage patients with mNSCLC on anti-EGFR and ALK inhibitors in Qatar. To our knowledge, this study is the first in the region that systematically assessed the above-mentioned objectives.

## Methods

This study is a descriptive retrospective cohort study, which was conducted in the NCCCR—the only cancer center in the state of Qatar, where NCCCR is a tertiary hospital for cancer patients under the umbrella of Hamad Medical Corporation (HMC) hospitals. The study covered patients treated either in the in-patient or out-patient settings from January 2015 to December 2019.

Inclusion criteria include all adult patients older than 18 years diagnosed with mNSCLC and had detected targetable EGFR or ALK mutations, who were started on at least 1 TKI either ALK or EGFR namely: afatinib, alectinib, crizotinib, erlotinib, gefitinib, and osimertinib. Patients might take more than 1 TKI over their course of therapy; each encounter was included. Previous history of chemotherapy and immunotherapy were allowed. However, immunotherapy was not previously used for any of the cases included in the study.

To identify the alteration genes among our population, polymerase chain reaction (PCR)-based methods was done for all patients on diagnosis with NSCLC, and NGS were used to detect specific resistance-related mutations for patients who progress during the treatment with TKIs.

Treatment clinical outcome of TKIs use was assessed using Response Evaluation Criteria in Solid Tumors (RECIST). The RECIST is a standardized system used to evaluate the response of solid tumors to treatment. It provides a consistent and objective way of measuring the change in size of a tumor or the appearance of new lesions after treatment.^14^ Physicians’ notes and radiological reports were also reviewed and assessed to identify clinical response to treatment. The safety profile was evaluated and recorded after examining adverse drug event (ADE) reports documented in patients’ profiles by the treating physicians or the clinical pharmacists. The severity of adverse events was measured and recorded using the National Cancer Institute’s Common Terminology Criteria for Adverse Events (NCI-CTCAE).

Data were extracted from electronic medical records (eMRs) called CERNER. The medical records were studied from initiation of TKIs to discontinuation either due to disease progression (DP), intolerance to TKIs, loss of follow-up, or patient’s death. Patients’ characteristics (eg, age, sex, ethnicity, smoking status), clinico-pathological characteristics and biological parameters (eg, mutation gene expressions, staging, brain metastasis, etc), medication details (eg, targeted therapy used, dose modification, switching to other medications, etc) and follow-up outcome measures (eg, response to therapy [complete response (CR), partial response (PR), stable disease (SD), or DP], patient status [died, lost follow-up], reported ADEs [grading of side effect per CTCAE criteria and presenting signs and symptoms], etc) were collected and analyzed. The objective response rate (ORR) was calculated as the total number of cases with CR or PR on any type of TKI over the course of therapy.

### Statistical analysis

Descriptive analysis was used to summarize descriptive data. For the normally distributed data, the results were reported with mean and standard deviation (SD); whereas median and inter-quartile range (IQR) were reported for skewed or non-normal data distribution. Categorical data were summarized using frequencies and percentages. The normality of the continuous data variable distribution was assessed using the Shapiro-Wilk test. Associations between 2 or more qualitative variables were assessed using the chi-square (χ^2^) test, the Fisher exact, or the Yates-corrected chi-square tests as appropriate. Quantitative data and outcome measured between the 2 independent groups (EGFR and ALK) were analyzed using unpaired *t*-test (or Mann-Whitney *U*-test for skewed data). One-way analysis of variance (ANOVA) followed by an appropriate post hoc test and chi-square tests was applied to compare various quantitative and qualitative parameters (such as age, sex, patient response, patient status, ethnicity, brain metastasis, gene mutation, switching to other medications, dose modification, smoking, and duration of treatment) across 4 groups namely: CR, PR, SD and DP. The ORR as descriptive data was estimated as the sum of CR, PR in percentages. All statistical analyses were done using statistical packages SPSS 28.0 (IBM Corp, Armonk, New York) and Epi-info (Centers for Disease Control and Prevention, Atlanta, Georgia) software.

## Results

A total cohort of 63 patients with mNSCLC harboring targetable mutation were identified during the study period of 5 years. Among these patients, 57.1% (36 of 63) expressed EGFR mutation and 42.9% (27 of 63) expressed ALK rearrangement. The diagnosis was more common in males than in females with 69.8% (44 of 63) vs 30.2% (19 of 63), respectively. The mean age at time of diagnosis was 66 years in both sexes.

### Characteristics and clinical outcomes in patients received epidermal growth factor receptor alteration

The vast majority of EGFR mutation patients were non-smokers 69.4% (25 of 36). In the EGFR group, a variety of ethnic groups were represented; Middle Easterners 38.9% (14 of 36) made up the majority, followed by Asians 36.1% (13 of 36). Males were more likely to have the diagnosis than females were, 66.7% (24 of 36) vs 33.3% (12 of 36), respectively. Table 1 provides more details regarding the characteristics of patients.

**Table 1.** Characteristics of patients with concurrent EGFR alterations.

N = 36	
Age in years, mean (±SD) 66 (±12.6)	
Sex, % (n)	
Male	66.7% (24/36)
Female	33.3% (12/36)
Ethnicity, % (n)	
Middle East	38.9% (14/36)
Asian	36.1% (13/36)
African	22.2% (8/36)
Caucasian	2.8% (1/36)
Smoking, % (n)	
Smoker	30.6% (11/36)
Non-smoker	69.4% (25/36)
EGFR mutations	
T790M	19.4% (7/36)
L858R	25.0% (9/36)
Brain metastasis (at any point of review), % (n)	
No	58.3% (21/36)
Yes	41.7% (15/36)
Number of patients received each medication, n	
Erlotinib	22
Gefitinib	3
Osimertinib	15
Afatinib	9
Switched to other medications (at any point of review), % (n)	
Yes	52.8% (19/36)
No	47.2% (17/36)
Dose modification (at any point of review), % (n)	
Yes	19.4% (7/36)
No	80.6% (29/36)
Patient status (at any point of review), % (n)	
Alive	38.9% (14/36)
Died	52.8% (19/36)
Lost to follow-up	8.3% (3/36)

**Figure 1.**
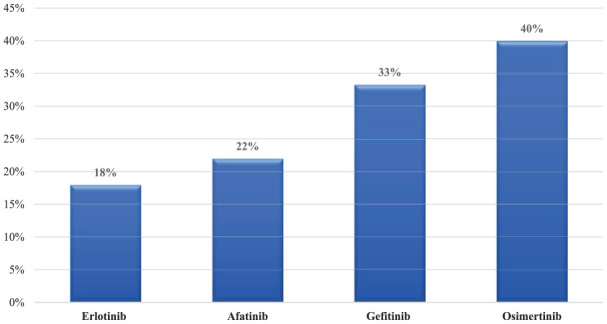
Objective response rate (ORR) for EGFR inhibitors.

Among the total population of EGFR mutation, 69.4% (25 of 36) were non-smokers at the time of diagnosis. On diagnosis, 19.4% (7 of 36) of patients had T790M molecular mutation and 25% (9 of 36) had L858R mutation detected. In addition, 41.7% (15 of 36) of the patients had central nervous system (CNS) metastasis either on diagnosis or progression. Erlotinib was the most commonly used EGFRi with 22 patients, followed by osimertinib 15 patients, and 11 patients received more than 1 EGFRi. The analysis showed that the overall CR rate to EGFRi was 19.4% (7/36). The detailed characteristics of patients with epidermal growth factor receptor (EGFR) mutation are described in Table 1. Moreover, ORR was variable among different anti-EGFR TKIs. The ORR was 40% with osimertinib, 33% with gefitinib and afatinib with 22%, followed by Erlotinib with 18%, as described in Figure 1.

**Figure 2.**
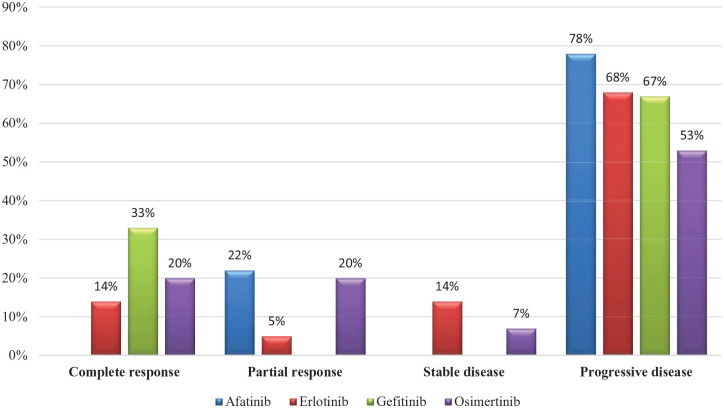
Response rate for EGFR inhibitors.

Similarly, to the ORR, the highest CR was seen in the osimertinib group. Although the highest rate of DP was in afatinib group (78%). The response rate details are described in Table 2 and Figure 2. Regarding the duration of response, the longest duration was in osimertinib group as 26 months, followed by gefitinib as 25 months, whereas the least duration was in afatinib group as 4 months. The mean duration of response for EGFR inhibitors is described in Table 3.

**Table 2.** Response rate for patient with EGFR mutation.

	Afatinib (N = 9)	Erlotinib (N = 22)	Gefitinib (N = 3)	Osimertinib (N = 15)
Complete response (CR)	–	14% (3/22)	33% (1/3)	20% (3/15)
Partial response (PR)	22% (2/9)	4% (1/22)	–	20% (3/15)
Objective response rate (CR + PR)	22% (2/9)	18% (4/22)	33% (1/3)	40% (6/15)
Stable disease	–	14% (3/22)	–	7% (1/15)
Progressive disease	78% (7/9)	68% (15/22)	67% (2/3)	53% (8/15)

**Table 3.** Duration in months of treatment in months for EGFR inhibitor group.

Medication	Median (months)	Number
Osimertinib	20	15
Gefitinib	14	3
Erlotinib	10	22
Afatinib	2	9

The most detected mutation in the EGFR cohort was Exon 19 deletion (44.4% [16/36]), followed by Exon 21 L858R (25% [9 of 36]). However, non-classical mutations were also detected in 8.3% (3 of 36) of patients and were presented in the group of patients who received 22.2% (2 of 9) afatinib and then osimertinib 13.3% (2 of 15). Most patients who received osimertinib were those patients who progressed on multiple lines of TKIs with high expression of molecular mutations (for more details, see Table 4).

**Table 4.** Mutations detected in patient with EGFR positive gene.

	N = 36				
	Exon 19 (N = 16)	Exon 21 L858R (N = 9)	T790M (N = 7)	Non-classical^a^ (N = 3)	No NGS (N = 8)
Afatinib	33.3% (3/9)	22.2% (2/9)	33.3% (3/9)	22.2% (2/9)	22.2% (2/9)
Erlotinib	40.9% (9/22)	22.7% (5/22)	18.1% (4/22)	–	31.8% (7/22)
Gefitinib	66.7% (2/3)	–	33.3% (1/3)	–	33.3% (1/3)
Osimertinib	53.3% (8/15)	26.7% (4/15)	40.0% (6/15)	13.3% (2/15)	6.7% (1/15)
	44.4% (16/36)	25% (9/36)	19.4% (7/36)	8.3% (3/36)	22.2% (8/36)

a Non-classical mutations: ERBB2 L 755P mutation, L718Q, and TP53 Q144.

### Characteristics and clinical outcome in patients used anaplastic lymphoma kinase rearrangement

Across patients with ALK mutation, most patients were non-smokers (66.7% [18 of 27]). Different ethnicities were presented in the study, the majority were Asians (44.4% [12 of 27]), followed by Middle Eastern (29.7% [8 of 27]). The diagnosis was more common in the males than in females (74.1% [20 of 27] vs 25.9% [7 of 27]), respectively. More information about patients’ characteristics is illustrated in Table 5.

**Table 5.** Characteristics of patients with concurrent ALK alterations.

N = 27	
Age in years, mean (±SD) 66 (±12.6)	
Sex, % (n)	
Male	74.1% (20/27)
Female	25.9% (7/27)
Ethnicity, % (n)	
Asian	44.4% (12/27)
African	22.2% (6/27)
Middle east	29.7% (8/27)
Caucasian	3.7% (1/27)
Smoking status, % (n)	
Smoker	33.3% (9/27)
Non-smoker	66.7% (18/27)
Brain metastasis (at any point of review), % (n)	
Yes	48.1% (13/27)
No	51.9% (14/27)
Patient status (at any point of review), % (n)	
Alive	63% (17/27)
Died	25.9% (7/27)
Lost to follow-up	11.1% (3/27)
Patient’s received each medication, n	
Alectinib	7
Crizotinib	25
Received both medications	5
Dose modification, % (n)	
Yes	25.9% (7/27)
No	74.1% (20/27)
Switched to other medications, % (n)	
Yes	33.3% (9/27)
No	66.7% (18/27)

In ALK-rearranged NSCLC group, patients received anaplastic lymphoma kinase inhibitor (ALKi) of either alectinib, crizotinib or both during their treatment period. In addition, 25 patients were started on crizotinib as first-line ALK inhibitor and five patients received alectinib as second line. The basic characteristics of the included ALK patients are presented in Table 5.

**Figure 3.**
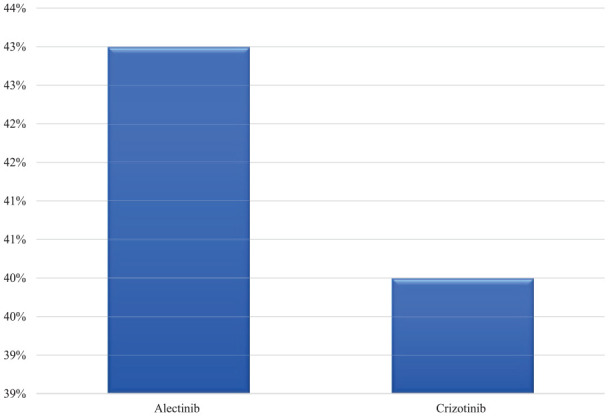
Objective response rate (ORR) for ALK inhibitors.

**Figure 4.**
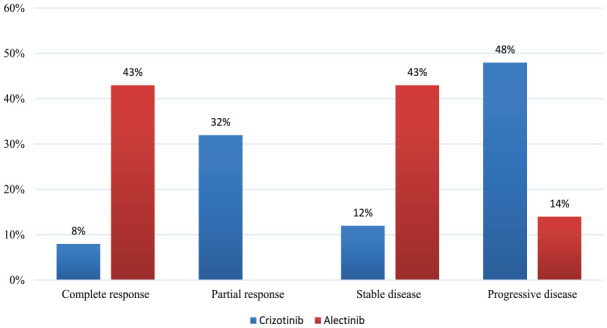
Response rate for ALK inhibitors.

The efficacy of ALK inhibitors was reported as ORR, for alectinib, it was 43%, and for Crizotinib, it was 40%, as seen in Figure 3. Similarly, the CR was higher with alectinib vs crizotinib as 43% vs 8%. Therefore, we observed higher rate of DP with crizotinib (48% vs 14%) (for more details, see Table 6 and Figure 4). The mean duration of treatment was longer with alectinib vs crizotinib as 16 vs 12.5 months (see Table 7).

**Table 6.** Response rate for patients with ALK alterations.

	Crizotinib (N = 25)	Alectinib (N = 7)
Complete response (CR)	8% (2/25)	43% (3/7)
Partial response (PR)	32% (8/25)	–
Objective response rate (CR + PR)	40% (10/25)	43% (3/7)
Stable disease	12% (3/25)	43% (3/7)
Progressive disease	48% (12/25)	14% (1/7)

**Table 7.** Duration of treatment in months for ALK inhibitor group.

Medication	Median (months)	Number
Alectinib	13	7
Crizotinib	9	25

### Safety

A total of 112 ADEs were documented in both EGFR and ALK groups—distributed as 71 ADEs in the EGFR cohort and 41 ADEs in the ALK cohort. The details of ADEs are shown in Table 8.

**Table 8.** The details of the reported ADEs.

ADEs reported with each ALK	ADEs reported with each EGFR inhibitor
• Alectinib CTCAE grade 3 to 4 occurring ADR: - Fever - Dyspnea - Lower limp swelling - Cough - Increased aminotransferase CTCAE grade 1 to 2 occurring ADR: - Elevated serum creatinine - Pneumonitis - Pleural effusion	• Erlotinib CTCAE grade 3 to 4 occurring ADR: - Diarrhea - Weakness CTCAE grade 1 to 2 occurring ADR: - Anemia - Fatigue - Fever and chills - Mouth ulcer - Poor appetite - Sepsis - Skin toxicity
• Crizotinib CTCAE grade 3 to 4 occurring ADR: - Abnormal menstruation - Candida esophagitis - Constipation - Visual disturbances - Dizziness - Increased Aminotransferase - Headache - Nausea and vomiting - Pulmonary embolism - Pneumonitis - Poor appetite - Skin rash - Renal impairment - Weight loss CTCAE grade 1 to 2 occurring ADR: - Ageusia - Bradycardia - Edema - Fatigue	• Osimertinib CTCAE grade 3 to 4 occurring ADR: - Acute kidney Injury - Bone pain - Cough - Drug-induced eczema - Increased aminotransferase - Fatigue - Fever - Gastric pain - Generalized body pain - Pharyngitis - QT prolongation - Thrombocytopenia - Xerosis cutis CTCAE grade 1 to 2 occurring ADR: - Abnormal cramps - Difficulty in swallowing - Dry cough - Skin toxicity - Hemoptysis - Index nail bed swelling - Nosebleed - Pneumonia - Productive cough - Rash - Rhinorrhea - Dyspnea
	• Afatinib CTCAE grade 1 to 2 occurring ADR: - Skin toxicity - Diarrhea

**Figure 5.**
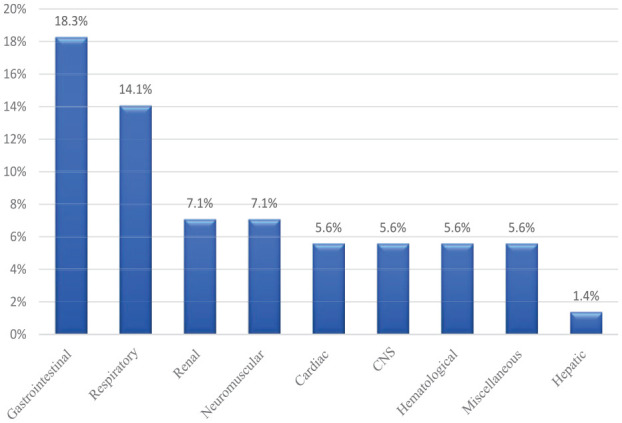
Percentage of ADEs associated with EGFR inhibitors.

In EGFR inhibitors cohort, the most common ADE was dermatological toxicity with an incidence of 30%, whereas hepatic toxicity with (1%) was the least frequent ADE. Remarkably, 5.6% of the patients developed cardiac toxicity on therapy that required dose adjustment, including QT prolongation, which was more common among osimertinib patients. More details on ADEs reported with EGFRi are shown in Figure 5.

**Figure 6.**
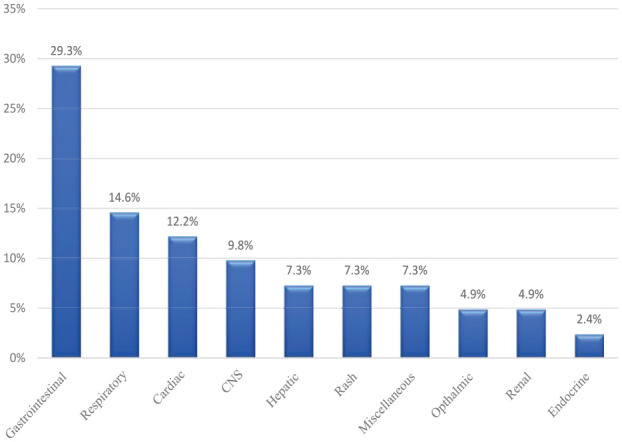
Percentage of toxicity associated with ALK inhibitors.

Focusing on the ALKi group, the most common ADE was gastrointestinal (GI) side effects; it affected 29% of the cases. The majority were documented with the use of crizotinib, where 43% of the patients stopped the medication due to the GI symptoms. On the contrary, the endocrine toxicity (2%) was the lowest. The respiratory toxicity accounted for second frequent event with (15%), presented mostly as cases of pneumonitis, where it was less severe with alectinib than in crizotinib-treated patients. Figure 6 lists the ADEs associated with anti-ALK TKIs.

## Discussion

The incidence of cancer, including NSCLC, is increasing worldwide and in the Middle East. This is mostly because of prolonged life expectancy and adoption of unhealthy life style.^15^ As a result, the need to focus on cancer research activities is noticeably increasing in the Middle East, with the view of understanding the treatment strategies implemented and comparing the results with the international experience.^16^ It is important to note that, in the State of Qatar, cancer research is growing rapidly and is making important strides. Researchers in Qatar have been actively involved in addressing a general lack of cancer research in the Middle East.^15,17^ The differences in genetics, demographics, environmental factors, and health care systems question the applicability of the mostly Western society–based medical research in other parts of the globe.^18^ Therefore, evaluating national research findings with international experience is an important step in identifying gaps in knowledge and areas where further research is needed. This can help guide future research priorities and collaborations between researchers from different countries.^19^-^21^

### Epidermal growth factor receptor inhibitor tyrosine kinase inhibitors

Patients with mutations in the EGFR gene can be detected with other mutations. Exon 19 deletion mutations account for 45% of mutations, whereas Exon 21 mutations, resulting in L858R substitutions, account for 40% to 45% of mutations that predict sensitivity to EGFR TKIs (like erlotinib, afatinib, gefitinib, and osimertinib) to slow or halt cancer cell growth. The remaining 10% of mutations involve Exon 18 and 20.^22-24^ The latter can be therapeutically inhibited by Food and Drug Administration (FDA)–approved mobocertinib. Partially activated mutant EGFRs can be rendered fully ligand-independent, and therefore constitutively active, by second-site substitutions in EGFR, such as the T790M mutation in Exon 20, which can also be targeted by osimertinib.^8,25^

Published data of the T790M mutation reflect an international rate of 0.32% to 78.95% in EGFR-TKI-naïve patients.^26^ However, the rate with EGFR-TKI-refractory patients is 50% to 60%.^27^ Nevertheless, the rate of T790M mutations among our EGFR-TKI-refractory patients was 19.4% of the cohort, which is lower that the international incidence. This might be due to the fact that, not all of our patients had NGS done because of the unavailability of the test earlier.

### Osimertinib

In both randomized clinical trials (RCTs) and Real-World Evidence (RWE), osimertinib has shown an ORR ranging from 50% to 71% in patients with stage 4 NSCLC who have EGFR with Exon 19 deletion, T790M, or Exon 21 mutation with L858R substitution mutations and have received prior treatment with EGFR TKIs such as erlotinib or gefitinib. For example, a retrospective study of 77 patients with stage 4 NSCLC who received osimertinib after previous treatment with EGFR TKIs reported an ORR of 64%.^28-31^

In our population, osimertinib was used as an advanced line of therapy after failure of other TKIs in 15 patients of EGFR mutant cases, with survival rate of 64.7%, median duration of response of 20 months, and ORR of 40% which is relatively close to the published data from clinical trial and RWE. Nevertheless, 7% (1 of 15) of the patient had SD while on osimertinib. Notably, 53.3% (8 of 15) of positive responders had Exon 19 deletion detected mutation, with or without T790M mutation detected, whereas 26.7% (4 of 15) of cases had Exon 21 L858R substitution. In addition, it is worth to mention that 2 of 15 cases of patients progressed on osimertinib had non-classical mutations—one of them had EGFR mutation, L718Q, and TP53 Q144 detected, whereas the other one had ERBB2 L 755P mutation.

### Gefitinib

The ORR in our study for gefitinib was 33%. The most well-known phase III clinical trial involving gefitinib was the Iressa Pan-ASia Study (IPASS) Study, which demonstrated the efficacy of gefitinib in patients, with an ORR for gefitinib of around 71.2%, which was significantly higher than the response rate in patients treated with standard chemotherapy.^32-34^ However, RWE shows lower ORR response rate to gefitinib of around 62%, which is still significantly higher than our results.^35-37^

### Erlotinib

Our study showed a 50% survival rate, an ORR of 18% to erlotinib, and 14% with SD. However, in several phase III clinical trials, erlotinib has shown ORRs ranging from approximately 60% to 80% in patients with EGFR mutation-positive advanced NSCLC who had not received previous treatment with an EGFR TKI.^36^ For example, a phase III clinical trial comparing erlotinib to chemotherapy as a first-line treatment in patients with EGFR mutation-positive advanced NSCLC reported an ORR of 65% in the erlotinib group.^37^ However, other studies showed that in patients with EGFR mutation-positive advanced NSCLC who had previously received treatment with chemotherapy, the ORR to erlotinib has been reported to range from approximately 7% to 11%.^38^ These findings might justify our relatively low percentage as most of our patients received prior therapy.

Erlotinib was not available as formulary drug in Qatar from 2013 to 2015. Therefore, it was not used as first-line therapy. Among our population, erlotinib exceptionally requested as non-formulary drug for 20% (2 of 10) of cases, after progression on the available lines of therapy. Thus, at the time, it was introduced to formulary in 2016; it was used as first line in 80% (8 of 10) of the cases. These facts might justify our relatively low overall ORR percentage.

### Afatinib

In clinical trials, afatinib has shown an ORR ranging from 50% to 70% in patients with EGFR mutation-positive advanced NSCLC who had not received previous treatment with an EGFR TKIs.^39^ For example, in Wu YL et al study, the phase III clinical trial comparing afatinib to chemotherapy (as a first-line treatment in advanced NSCLC patients with EGFR mutation-positive) reported an ORR of 56% in the afatinib group.^40^ In patients with EGFR T790M mutation-positive advanced NSCLC who had previously received treatment with an EGFR TKIs, the ORR to afatinib has been reported to range from approximately 28% to 32%.^39,41,42^ Several real-world studies have reported ORRs ranging from approximately 30% to 60% in patients with EGFR mutation-positive advanced NSCLC who received afatinib as a first-line or subsequent-line treatment.^43,44^ For example, a retrospective study of 232 patients with EGFR mutation-positive advanced NSCLC who received afatinib reported an ORR of 50%.^42^ On the contrary, patients who received afatinib as second line had ORR of 22.7%,^43^ whereas those who received it in the rechallenge setting after DP or had been discontinued due to side effects of one of first-generation TKIs had ORR 25%.^44^ In our population, afatinib cohort showed 22% ORR, which is close to the internationally reported values in second or subsequent lines. And this can relate to the fact that most of our patients received it as an advanced line of therapy.

As per the above-mentioned results, there is a consistent low ORR among our population to EGFRi as compared with the international values, which reflects the need for prospective studies to further study the specific characteristics of our population and factors that might impact the clinical response to EGFRi.

### Anaplastic lymphoma kinase inhibitor tyrosine kinase inhibitors

Similar to anti-EGFR TKIs, anti-ALK-TKIs are small molecules that enter the cell and bind to the intracellular domain of ALK receptors to inhibit transphosphorylation, and therefore the downstream pro-proliferation signaling.^11,45^

### Crizotinib

In our population, crizotinib had an ORR of 40% and 12% had SD. The PROFILE 1014 trial, a pivotal phase III clinical trial, compared the efficacy of crizotinib to standard chemotherapy in previously untreated ALK-positive advanced NSCLC patients. The trial reported that crizotinib had a significantly higher ORR as compared with chemotherapy, 74% (95% CI = 67-81) for crizotinib vs 45% (95% CI = 37-53) for chemotherapy.^46^ Whereas, RWEs showed an ORR of 58% which is lower than the PROFILE 1014 trial, but close to our results.

### Alectinib

Alectinib is another example of ALK inhibitors, which have been approved for the management of ALK-positive mNSCLC. The ALEX trial is a key phase III clinical trial published in the *New England Journal of Medicine* in 2017, comparing alectinib to crizotinib, in therapy-naïve patients with ALK-positive advanced NSCLC. The results of ALEX trial demonstrated that alectinib had a significantly higher overall response rate (ORR) compared with crizotinib. The ORR for alectinib was 82.9%, whereas the ORR for crizotinib was 75.5%.^46,47^ Adding to a real-world study conducted in Japan assessing the effectiveness and safety of alectinib in patients with ALK-positive NSCLC who were previously exposed to crizotinib. The study reported an ORR of 65.6% for alectinib, which is higher than our results presented in this study of 43% ORR of alectinib,^48^ in addition to 43% of the patient had SD.

### Safety

#### Antiepidermal growth factor receptor tyrosine kinase inhibitors

The published data show some variations in the incidence rate of adverse events of anti-EGFR TKIs in different clinical trials. However, the overall incident rates are commonly reported as follows: skin rash (50%-90%), diarrhea (30%-60%), fatigue (20%-40%), nausea and vomiting (20%-30%), and paronychia (10%-30%).^49^ Our study showed relatively lower rate of adverse events, with skin side effects of 30%, followed by GI side effects of 10%. A possible explanation for the lower rate of ADEs in our sample compared with the published data is the lower mean age in our population of 66 years compared with 72.8 years.

#### Antianaplastic lymphoma kinase tyrosine kinase inhibitors

The published data regarding the ADEs incident rates commonly reported with anti-ALK TKIs are GI, peripheral edema, and respiratory events, 30%, 20%, and 5%, respectively.^50,51^ Our study results showed that anti-ALK TKIs caused a range of toxicity, commonly GI followed by respiratory, with an incidence rate of 29% and 15%, respectively. Noticeably, the respiratory ADEs among our population are higher as compared with the published data.

### Limitations

It is important to note that being a retrospective study, this research design inherently possesses limitations, such as the potential for selection bias and limited control over confounding factors. Furthermore, baseline performance status was not found for most of the patients in the medical records. To minimize the selection bias, and to come over the limitation of the small sample size, we included all mNSCLC patients who received TKIs that are existing in our records till the time of study and tried to include the confounding factors in the analysis. But we could not correlate the different ethnicities to the ORR of each medication, which may require further studies to have such results. Moreover, the study’s reliance on medical records lacks information about performance status and data from previous treatments, which may introduce inherent inaccuracies. This may reflect on another confounder, suggesting the possibility of alteration in the safety profile imposed by the impact of prior treatments. It is possible that this had an impact on the patients’ general health or went so far as to interfere with the subsequent treatments. We attempted to overcome this by independently validating the negative effects of TKI use that have been reported and updating the information that is currently accessible regarding the use of prior medicines. Hence, the results should be interpreted with caution and warrant further investigation through prospective randomized controlled trials. Owing to the small sample size, the breakdown of side effects was very insignificant. Therefore, the authors decided to group them, to be more informative to the readers. However, we did mention the detailed side effects in Table 8 and Figures 5 and 6.

Baseline NGS was not available for all patients, as study included patients from 2015 when NGS was not available. This is another limitation of the study. Considering that NSCLC NGS panel is the standard of care nowadays, and that it also includes many mutations that were not done during the study period, further studies can be beneficial including more medication classes and alterations. However, in this study, we included all data and mutations that have been documented for the study cases.

## Conclusions

The findings of this study shed light on the benefits and risks associated with TKIs in the management of mNSCLC as a challenging disease in a multi-nationality population. The clinical outcome revealed results, indicating that TKIs have shown significant clinical activity in the management of mNSCLC in our reviewed cohort. The TKIs were generally well-tolerated by most patients, and the reported ADEs are well-known, including GI disturbances, dermatological issues, hepatotoxicity, and cardiovascular events. The study highlights the importance of close monitoring and management of these side effects to ensure the optimal balance between treatment efficacy and patient well-being. This study can be considered as an overview of practice in the management of mNSCLC using TKIs. However, to reflect comprehensively on the clinical outcomes, prospective comparative studies are highly needed, with strict application of robust guidelines.
